# Spontaneous Bilateral Hemothorax as a Case of Epithelioid Hemangioendothelioma (EHE)

**DOI:** 10.1155/2019/1854361

**Published:** 2019-02-24

**Authors:** Essa M. AlGhunaim, Hafsa AlNajem, Derar S. AlShehab

**Affiliations:** ^1^Thoracic and Foregut Surgery Unit, Chest Diseases Hospital, Kuwait; ^2^General Surgery Department, Mubarak Al-Kabeer Hospital, Kuwait

## Abstract

Epithelioid hemangioendothelioma (EHE) is a rare tumor that is vascular in origin, arising from the endothelial or preendothelial cells. It can occur at different sites of the body, commonly in the liver, lungs, and bone. Pulmonary EHE is usually symptomatic, sometimes presenting with a cough, dyspnea, or chest pain. Rarely, it presents with pleural effusion. Here, we are presenting a case of pleural epithelioid hemangioendothelioma presenting as a case of spontaneous bilateral hemothorax.

## 1. Introduction

Epithelioid hemangioendothelioma (EHE) is a rare vascular tumor of endothelial or preendothelial cell origin, and it represents less than 1% of all vascular tumors. EHE affects various sites of the body, but it typically affects the lungs and liver [1]. The presentations or manifestations of EHE are unspecific, and they vary according to the affected site. In this paper, we present a case of pleural EHE, which presented with bilateral spontaneous hemothorax. This case of EHE is reported due to its rare clinical presentation.

## 2. Case Report

**Figure 1:**
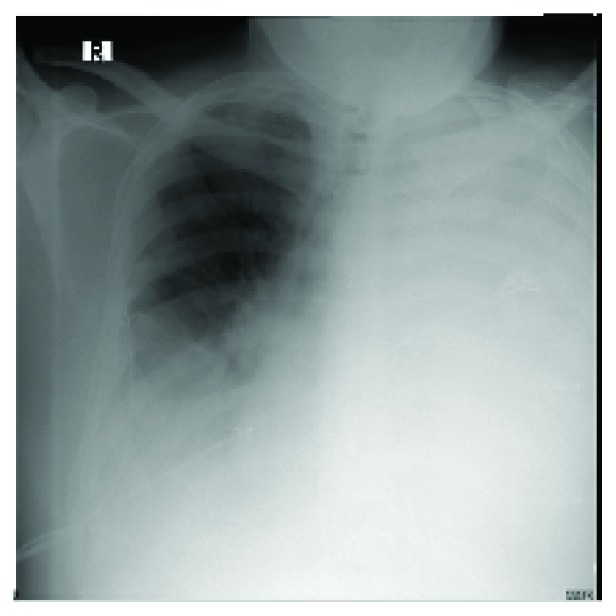
CXR of the case on admission showing the white out lung on the left side giving the impression of massive pleural effusion.

A 49-year-old male, a smoker of 15 packs/year, previously healthy, presented with a ten-day history of a sudden cough, associated with shortness of breath and left-sided chest pain. The patient also gave a history of loss of appetite and weight loss of about 15 kilograms for the past three months. On admission, his blood test revealed a drop in hemoglobin from 14.3 g/dL to 8.6 g/dL. The chest X-ray on admission showed bilateral lung opacity (Figure 1), and pleural tapping was done, which revealed a bloody content. Thoracic surgery service was consulted to evaluate the hemothorax.

**Figure 2:**
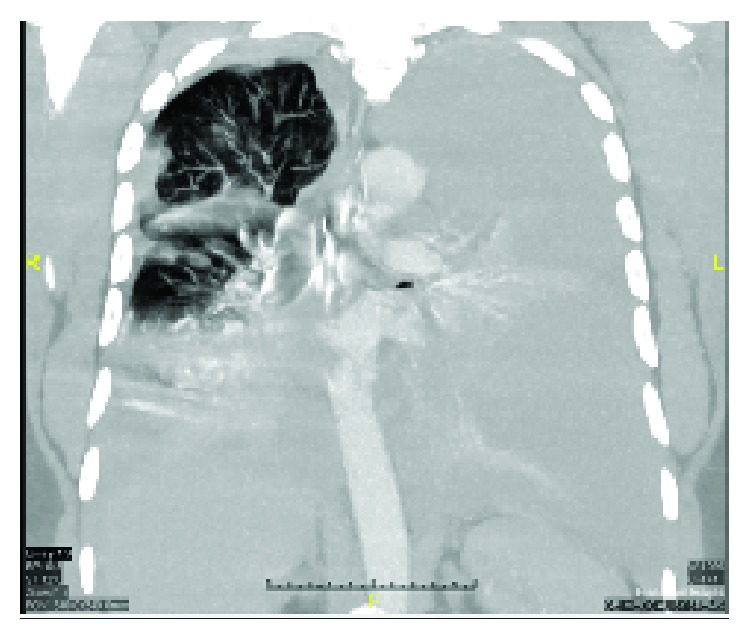
Initial CT chest of the patient (coronal view) with complete obliteration of the left hemithorax and some lung parenchyma seen in the right hemothorax. Hounsfield units were consistent with hemorrhagic effusion.

**Figure 3:**
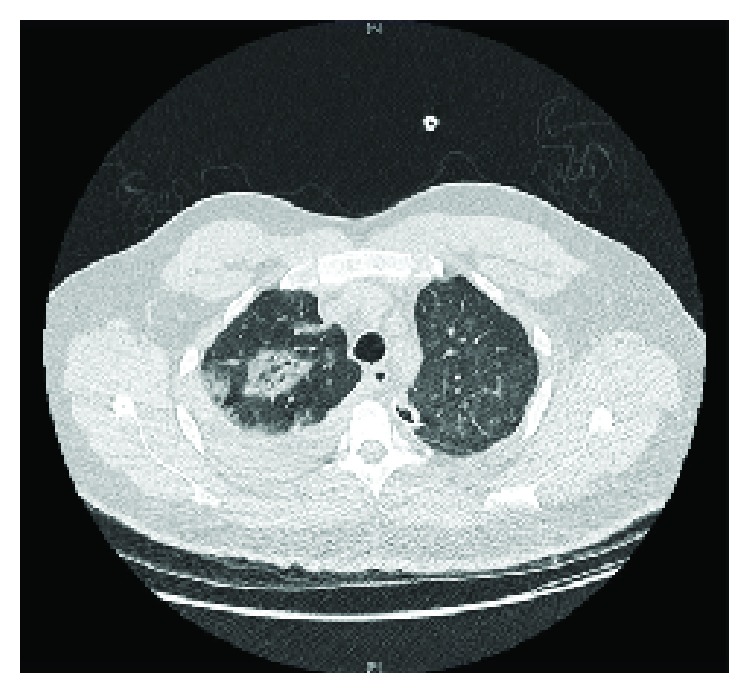
CT chest of the patient (axial view) showing the position of the chest tube and location of hemothorax.

**Figure 4:**
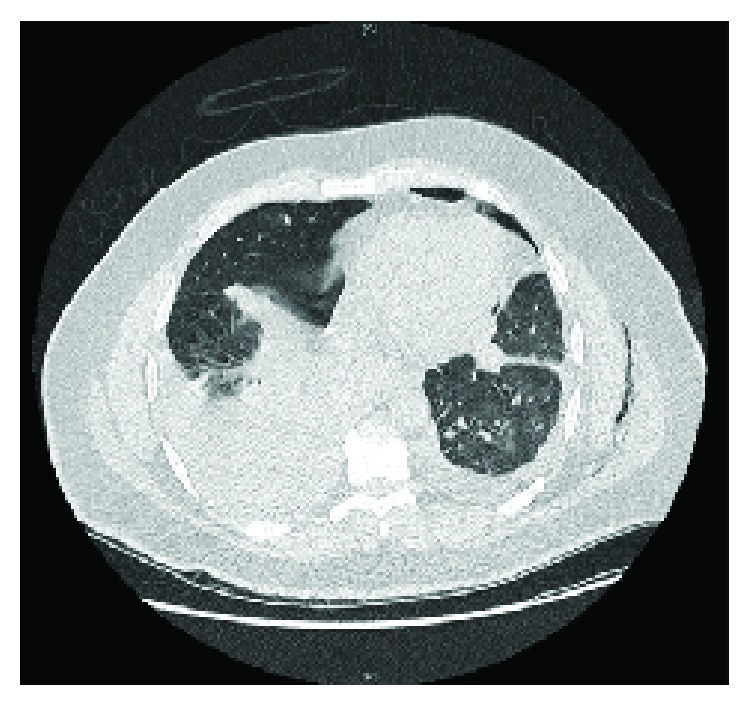
CT chest of the patient (axial view) showing the position of the chest tube and location of hemothorax.

**Figure 5:**
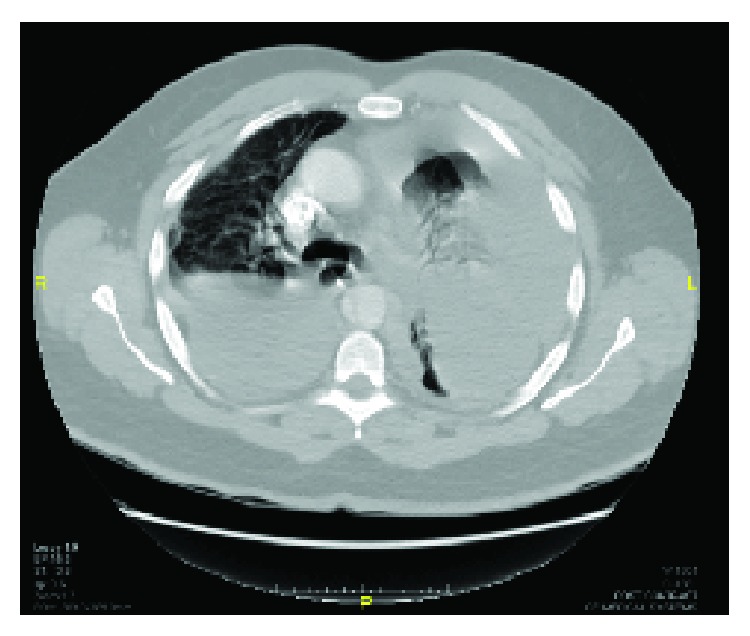
Another image of the CT chest (axial) in the lower chest showing cuts with massive hemothorax.

**Figure 6:**
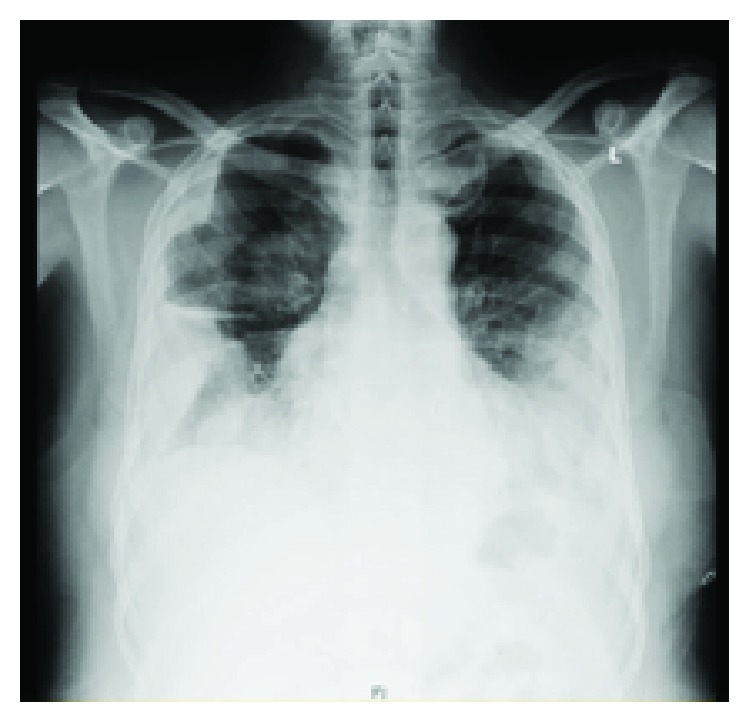
CXR postdrainage of hemothorax with abnormal pleura.

A CT scan of the chest with IV contrast was done, and it showed massive bilateral hemothorax, more on the left side causing lung collapse, but no evidence of contrast extravasations (Figures 2–5). Immediately, a chest tube (size: 28 Fr) was inserted in the left side, which yielded around 1000 mL of blood (Figure 6). The following day, the patient condition remained the same, and he was still complaining of shortness of breath and tachycardia. Thus, the patient was shifted under the care of thoracic surgery. A second chest tube (28 Fr) was inserted on the left side, and a pigtail was inserted on the right side. The right-sided pigtail yielded about 2700 mL of dark-colored blood. After the insertion of the previously mentioned tubes, the patient condition improved significantly, and the patient was stabilized. The following day, the patient underwent video-assisted thoracoscopy (VATS) pleural exploration and biopsy; it showed a nodular pleura and normal-looking lungs. Multiple biopsies were taken from the pleura and were sent for histopathology testing.

## 3. Pathological Findings

**Figure 7:**
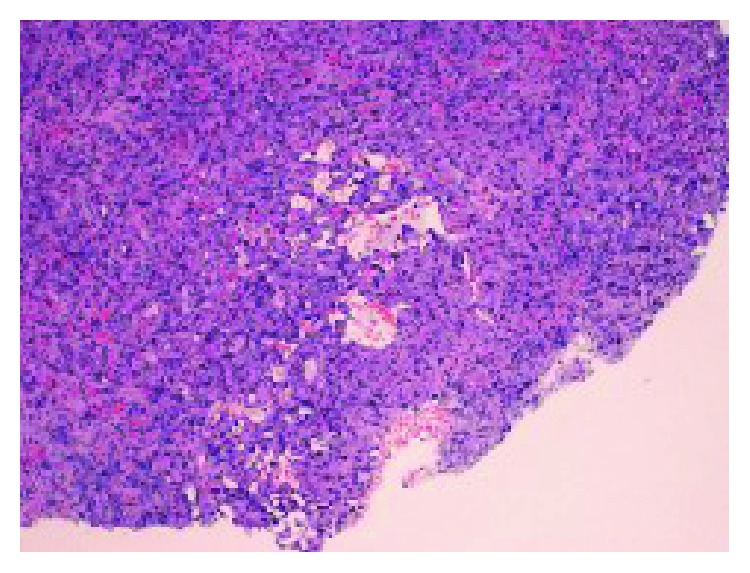
Histological photograph of the pleural specimen shows a pleural intraparenchymal tumor with focal irregular vascular spaces (H&E × 40x).

**Figure 8:**
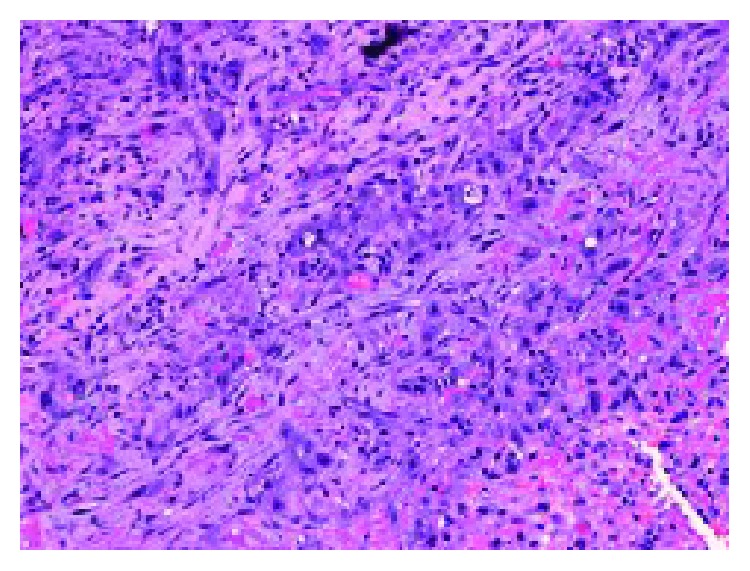
Cords and nests of epithelioid endothelial cells: some with intracytoplasmic vacuoles infiltrate the fibrous tissue (H&E × 100x).

**Figure 9:**
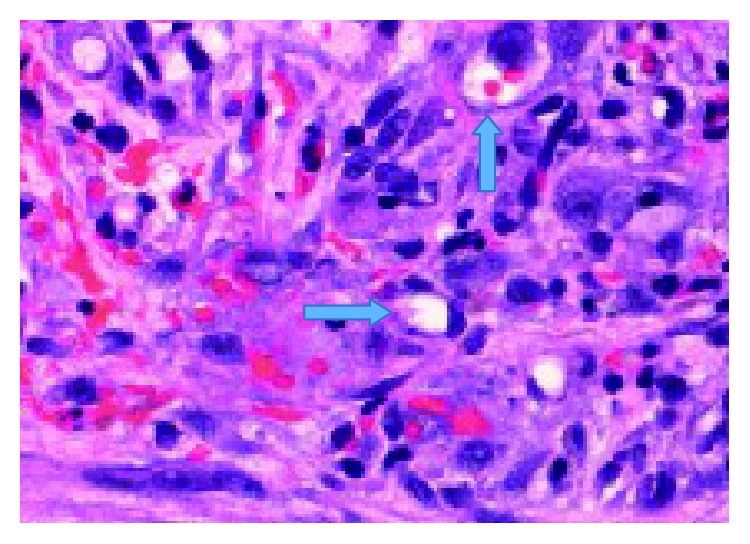
Epithelioid cells with pleomorphic nuclei: some with prominent nucleoli and intracytoplasmic vacuolization (arrows) (H&E × 400x).

**Figure 10:**
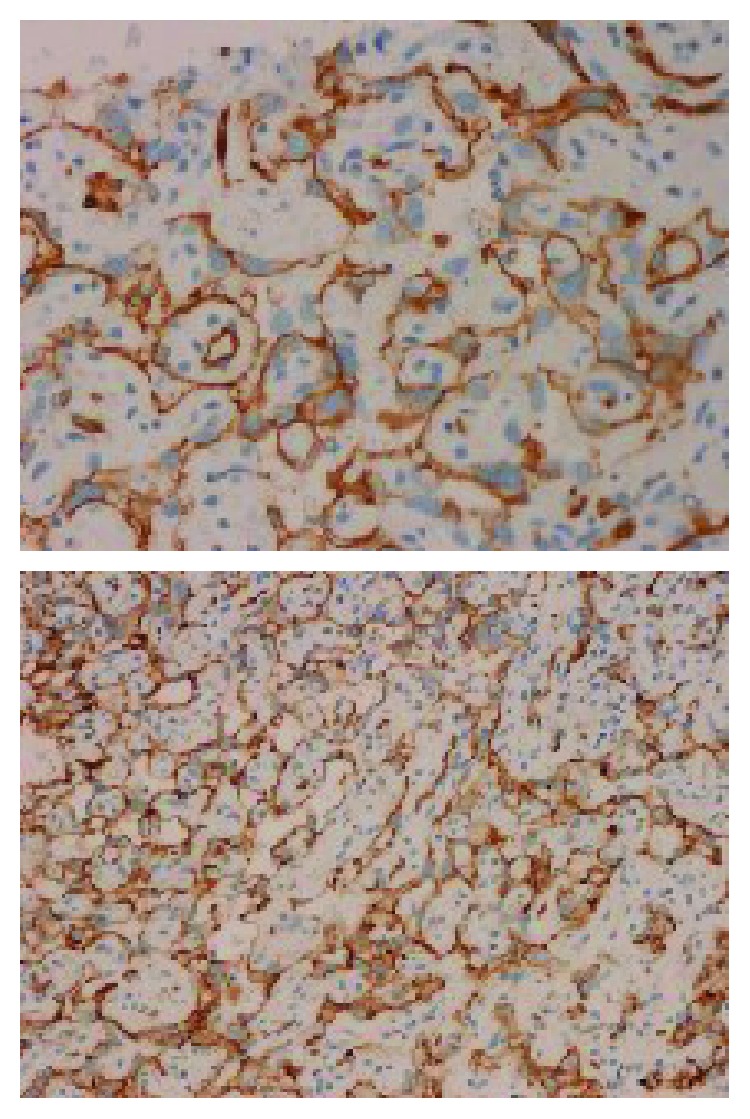
The CD34 and CD31 immunohistochemical stain shows positive reactivity in the tumor cells (×200).

Histopathological examination of the pleural biopsies revealed extensive infiltration of the pleura by atypical epithelioid cells arranged in clusters, in strands, or lying individually (Figures 7 and 8). The cells exhibit nuclear pleomorphism, mitosis, and intracytoplasmic vacuoles. There are irregular vascular channels lined by these atypical cells (Figure 9). The immunohistochemical stain showed positivity for CD34 and CD31 (Figure 10) but was negative for CK7, CK20, calretinin, TTF1, and napsin. It was consistent with epithelioid hemangioendothelioma.

The case was discussed in a multidisciplinary meeting, and the consensus is to start him on chemotherapy.

## 4. Discussion

Epithelioid hemangioendothelioma (EHE) is a rare vascular tumor of endothelial cell origin, with an estimated prevalence of less than one in 1 million [1, 2]. It was first described in 1975 by Dail and Liebow as an aggressive bronchoalveolar cell carcinoma [1]. EHE can involve a single organ or multiple organs simultaneously. The most commonly affected organs in EHE were the liver, lung, and bone. According to the observation and data analysis of Epithelioid Hemangioendothelioma and Related Vascular Disorders (HEARD) Support Group, the most common presentations for EHE were the liver alone (21%), liver plus lung (18%), lung alone (12%), and bone alone (14%) [3].

Histologically, the tumor is composed of small nests or short cords of rounded to slightly spindled endothelial cells that blend. A typical finding seen in immunohistochemistry is Weibel-Palade bodies seen in the cytoplasm of the endothelial cells [1]. In EHE, immunohistochemical stains for vascular endothelial markers are usually positive for CD31 and CD34 and negative for epithelial markers, like cytokines [4].

Fifty to seventy-six percent (50%-76%) of patients with pulmonary EHE are asymptomatic and are incidentally diagnosed by abnormal chest radiography [1]. EHE may present with nonspecific symptoms such as a cough, dyspnea, pleuritic chest pain, and hemoptysis. Pleural EHE may manifest with pleural effusion, thickening, or pleural tumor; most cases with pleural effusion were serosanguineous in nature [4]. Márquez-Medina et al. reviewed about 22 cases of EHE with pleural effusion, and most of the cases (around 90% of the cases) presented with pleural effusion which was serosanguineous in nature; only 2 of the reviewed cases presented with hemothorax [3, 4].

Pleural epithelioid hemangioendothelioma carries a poor prognosis with a mean survival of 10–12 months. Due to the rarity of this condition, there is still no standardized treatment.

## 5. Conclusion

Epithelioid hemangioendothelioma is a rare vascular tumor with poor prognosis; a bilateral spontaneous pleural hemothorax may well be the single presentation. Treatment of such condition is not adequate which may require further investigation.

## References

[1] Sardaro A., Bardoscia L., Petruzzelli M. F., Portaluri M. (2014). Epithelioid hemangioendothelioma: an overview and update on a rare vascular tumor. *Oncology Reviews*.

[2] Woodall C. E., Scoggins C. R., Lewis A. M., McMasters K., Martin R. C. (2008). Hepatic malignant epithelioid hemangioendothelioma: a case report and review of the literature. *The American Surgeon*.

[3] Ali H. A., Lippmann M., Khaleeq G. (2008). Spontaneous hemothorax secondary to epithelioid hemangioendothelioma. *Chest*.

[4] Photowala F., Hatipoglu U. S., Garcia C. (2012). An older patient with bilateral non-traumatic haemothoraces. *BMJ Case Reports*.

